# Donaldson’s Pulp-Canal Cleansers

**Published:** 1886-03

**Authors:** 


DONALDSON'S PULP-CANAL CLEANSERS.

For a long time we have used Donaldson's nerve-canal broaches, and have found nothing equal to them in temper and delicacy. Now he has introduced a new instrument for work in nerve canals. Very delicate, spirally cut barbs are made upon the broaches, and they may be advanced or retracted like a screw, thus avoiding the great danger of breakage, while their efficiency is increased. For the purpose intended they are the most perfect instruments that we have ever used.

